# Multivariable logistic and linear regression models for identification of clinically useful biomarkers for osteoarthritis

**DOI:** 10.1038/s41598-020-68077-0

**Published:** 2020-07-09

**Authors:** Yulia Liem, Andrew Judge, John Kirwan, Khadija Ourradi, Yunfei Li, Mohammed Sharif

**Affiliations:** 1 Translational Health Sciences, Bristol Medical School, Musculoskeletal Research Unit, Southmead Hospital, University of Bristol, Learning and Research Building (Level 2), Bristol, BS10 5NB UK; 2 University of Bristol, Biomedical Sciences Building, University Walk, Bristol, UK

**Keywords:** Osteoarthritis, Diagnostic markers, Predictive markers, Osteoarthritis

## Abstract

Osteoarthritis (OA) is the most common chronic degenerative joint disease which causes substantial joint pain, deformity and loss of activities of daily living. Currently, there are over 500 million OA cases worldwide, and there is an urgent need to identify biomarkers for early detection, and monitoring disease progression in patients without obvious radiographic damage to the joint. We have used regression modelling to describe the association of 19 of the currently available biomarkers (predictors) with key radiographic and clinical features of OA (outcomes) in one of the largest and best characterised OA cohort (NIH Osteoarthritis Initiative). We demonstrate that of the 19 currently available biomarkers only 4 (serum Coll2-1 NO2, CS846, COMP and urinary CTXII) were consistently associated with established radiographic and/or clinical features of OA. These biomarkers are independent of one another and provide additional predictive power over, and above established predictors of OA such as age, gender, BMI and race. We also show that that urinary CTXII had the strongest and consistent associations with clinical symptoms of OA as well as radiographic evidence of joint damage. Accordingly, urinary CTXII may aid in early diagnosis of OA in symptomatic patients without radiographic evidence of OA.

## Introduction

Osteoarthritis (OA) is a common chronic degenerative disease affecting the knees, hips, hands and spine. Recent data suggests that 40% of men and 47% of women over 25 are at risk of OA and over 250 million people are affected worldwide^1,2^. In the UK over 3 million GP consultations and 115,000 hospital admissions/year are due to OA and it costs the UK economy approximately £3.2 billion in lost working days alone^3^. OA is an increasing burden on the NHS and other providers of healthcare in the western countries. OA is a multifactorial disease of unknown aetiology. The major risk factors for developing OA are age, gender, obesity, injuries and joint shape. The disease develops slowly over the years and is characterized by progressive loss of articular cartilage, subchondral bone remodelling, osteophytes formation and variable synovial inflammation. Currently diagnosis is based on symptoms, and usually confirmed with X-ray at late-stage disease when there is already irreparable damage to the joints. Other imaging methods such as Magnetic Resonance Imaging (MRI) and Dual-energy X-ray absorptiometry (DXA) may be used for early diagnosis and monitoring OA but widespread routine use of these imaging techniques is limited by their availability and/or costs. Although MRI can be used early in OA and can be predictive of radiographic change as it can directly visualise all articular tissues^4,5^, it is not cost-effective and is time consuming^6^. Dual-energy X-ray absorptiometry (DXA) may be used to measure bone mineral density (BMD) at specific regions of interest (ROI) in OA as increased BMD is known to be associated with an increase in Kellgren and Lawrence (K&L) grade, sub-chondral sclerosis and minimum joint space width (JSW)^7^. A major limitation of DXA is that the position of the knee must be identical in serial readings for accurate data. Also, soft tissue swelling may prevent the full extension of the knee preventing the DXA image from being within the ROI^8,9^. Therefore, there is an urgent need for identification of suitable biomarkers for early detection and monitoring OA.

During the last three decades we and others have demonstrated that serum and urinary levels of biomarkers can provide a way of monitoring OA and some of these biomarkers (mediators and products of intra-artricular tissue degradation, attempted repair and inflammation) are clearly associated with biological and pathological processes in knee OA^10–13^. Yet none of the biomarkers have been shown to be sufficiently specific or sensitive for early diagnosis and/or monitoring of individual at-risk patients, and/or predict the course of disease with time^14^. Many research programmes aim to develop pharmaceutical treatments for OA that will alter the course of the disease, but these programmes are significantly hampered by the lack of good biochemical methods for early detection and monitoring of disease progression. The slow progress in the field is largely due to a lack of suitable cohorts and robust analytical tools for discovery of OA-specific biomarkers. The availability of clinical, biomarker and radiographic data from the National Institute of Health (NIH) Osteoarthritis Initiative (OAI) multi-centre prospective 96 months observational cohort provides an opportunity to identify clinically useful biomarkers for knee OA.

## Methods

**Figure 1:**
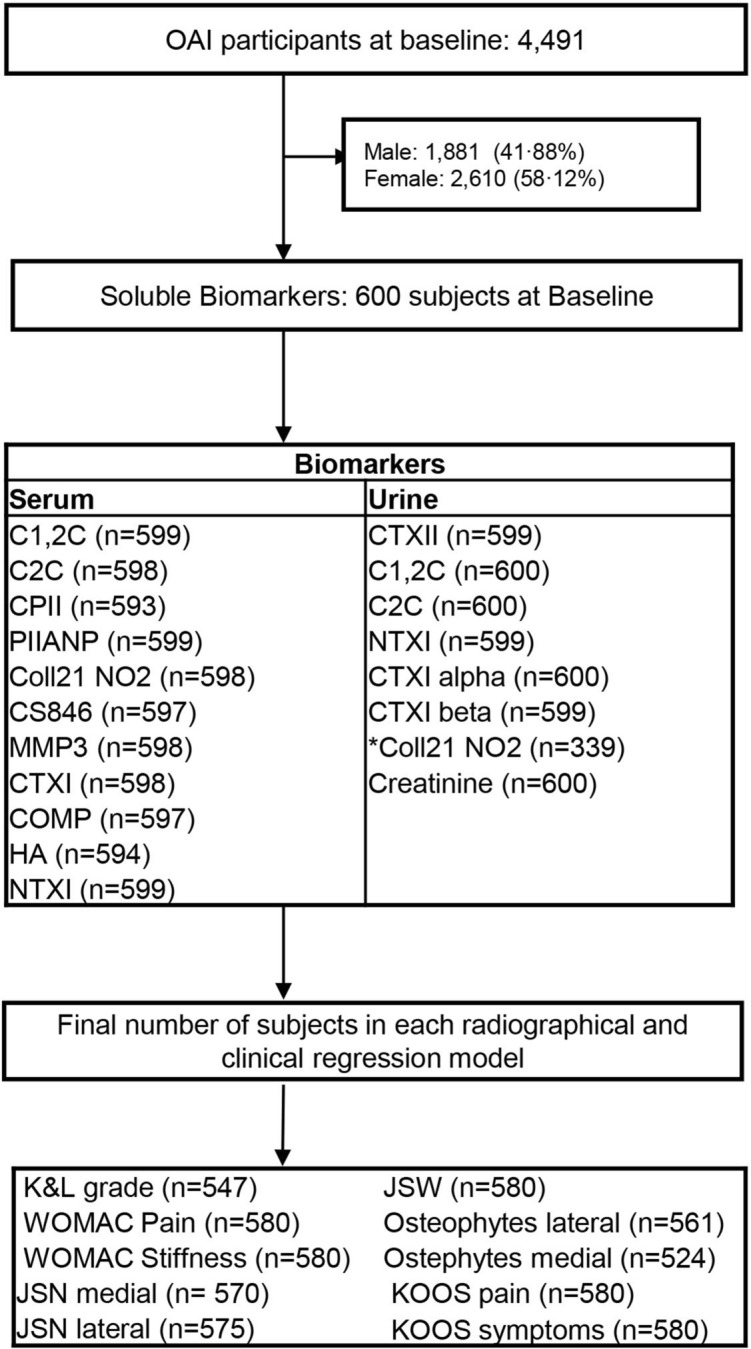
Study design: Total number of subjects for each biomarker, radiographic and clinical variables. *Urinary Coll 2-1 NO2 was removed due to large number of missing data.

All data were obtained from the Osteoarthritis Initiative (OAI) database (https://www.nia.nih.gov/research/resource/osteoarthritis-initiative-oai). OAI is a longitudinal cohort study sponsored by the National Institute of Health (NIH) to understand how to prevent and treat knee OA^15^. The study recruited a total of 4,491 men and women aged from 45 to 79, with, or at risk of symptomatic femoral-tibial knee OA (supplementary Table 1). The study included all ethnic minorities but mainly focused on African Americans. Men and women with inflammatory arthritis (mainly rheumatoid arthritis, RA), contraindication to MRI and bilateral end-stage knee OA were excluded from the study. 4,491 subjects were followed up annually for 10 years and demographic, clinical, radiographic, blood and urine samples were collected at baseline and at yearly follow up. The details of the study protocols and all informed patient consent documentation were reviewed and approved by the OAI-NIH (https://oai.epi-ucsf.org/datarelease/docs/StudyDesignProtocol.pdf)^15^. Our study is a retrospective study using publicly available data from a subset of 600 OAI patients who were K&L grade 1 or more and had biochemical markers measured at baseline, 12 and 24 months in blood and/or urine (Fig. 1).

**Table 1:** Multivariate logistic regression model for biomarkers showing associations with either radiographic and/or clinical features of OA.

Outcome	sColl2-1 NO2		sCS846		sCOMP		uCTXII	
	OR (95%)	p-value	OR (95%)	p-value	OR (95%)	p-value	OR (95%)	p-value
KL grade	**1.1316 (1.0289, 1.2445)**	**0.011**	0.9972 (0.9913, 1.0032)	0.364	**0.9988 (0.9977, 0.9999)**	**0.036**	**1.0607 (1.0231, 1.0996)**	**0.001**
WOMAC pain	1.0012 (0.9614, 1.0427)	0.954	1.0032 (0.9999, 1.0065)	0.056	**0.9991 (0.9984, 0.9997)**	**0.005**	**1.0138 (1.0023, 1.0253)**	**0.018**
WOMAC Stiffness	1.0137 (0.9710, 1.0584)	0.535	**1**.**0048 (1.0011, 1**.**0085)**	**0.011**	1.0005 (0.9999, 1.0012)	0.110	1.0072 (0.9954, 1.0192)	0.232
JSN medial	0.9967 (0.9511, 1.0446)	0.892	1.0014 (0.9976, 1.0053)	0.466	1.0002 (0.9994, 1.001)	0.699	**1.0164 (1.0013, 1.0318)**	**0.033**
JSN lateral	1.0704 (0.9967, 1.1495)	0.062	**0.9909 (0**.**9829, 0**.**9989)**	**0.026**	**1.0015 (1.0004, 1.0027)**	**0.008**	0.9918 (0.9692, 1.015)	0.484
Osteophytes medial	0.9860 (0.9168, 1.0605)	0.705	0.9957 (0.9906, 1.0007)	0.092	0.9998 (0.9986, 1.001)	0.699	1.023 (0.9965, 1.0502)	0.090
Osteophytes lateral	**1.0561 (1.0059, 1.1089)**	**0**.**028**	1.0007 (0.9971, 1.0042)	0.716	0.9997 (0.999, 1.0004)	0.431	**1.0205 (1.0061, 1.0351)**	**0.005**

Age, BMI, gender, and race were adjusted for in the model.

Statistically significant data are shown in bold.

### Biomarkers

All available biomarker data at baseline, 12 and 24 months from the subsets of 600 patients (Fig. 1) were extracted from the OAI data set. The biomarkers that were analysed in serum included C-propeptide of type II collagen (CPII), oligomeric matrix protein (COMP), N-propeptide of collagen IIA (PIIANP), chondroitin sulphate 846 (CS846), C-terminal cross-linked telopeptide of type I collagen (CTXI), hyaluronic acid (HA), matrix metalloproteinase-3 (MMP-3), types I and II collagen marker degradation markers (C1,2C, C2C), the nitrated form of α-helical region of type II collagen (Coll2-1 NO2) and the cross-linked N-telopeptide of type I collagen (NTXI). Biomarkers analysed in urine were C-terminal crosslinked telopeptide of type II collagen (CTXII), and alpha and beta isomerised versions of the CTXI (CTXIα `and CTXIβ). The protocol for collection, processing of the blood samples, selection and quantification of the biomarkers had been described elsewhere^15^.

### Radiographic features of OA

The radiographic features of OA investigated included joint space narrowing (JSN) or joint space width (JSW), osteophytes and Kellgren and Lawrence (K&L) grade which indicate the overall radiographic severity of knee OA. These radiographic data from baseline and all follow up visits from 600 patients who have biomarker measurements were obtained from the OAI Website.

### Clinical features of OA

The clinical features of OA investigated included joint pain and stiffness. We downloaded the Western Ontario and McMaster Universities Osteoarthritis Index (WOMAC). The WOMAC index (set of standardized questionnaires) is commonly used by health professionals to evaluate the condition of patients with OA of the knee, and includes pain, stiffness, and physical functioning of the joints. The Knee injury and Osteoarthritis Outcome Score (KOOS) is an extension of the WOMAC index but is considered to be more sensitive and responsive than the WOMAC in younger or more active patients^16,17^. Since pain is the main symptom of OA, we focused on WOMAC and KOOS pain in this study. WOMAC and KOOS pain scores are available from baseline and follow up visits which were downloaded from the OAI website. Baseline demographic information such as age, race, gender and BMI were also obtained.

### Data cleaning and descriptive statistics

Data cleaning for all biochemical markers, radiographic and clinical measurements were performed using Stata 15.1. The mean, standard deviation and the frequencies for each variable were then calculated. The biomarker (continuous data) were examined and any outliers were identified and removed by using the cut off range available from Kraus et al.^18^. The dataset contains information on outcome (radiographic and clinical variables) for both left and right knees. The biomarkers are collected at a person level and are not knee specific. We chose to do a patient-based analysis using the maximum K&L grade for the worst knee for each patient.

### Statistical analysis

Spearman correlation was used to evaluate multicollinearity of 19 biochemical biomarkers. Serum NTXI, urinary NTXI, urinary CTXI alpha and urinary CTXI beta were removed due to strong correlation with each other in the final model. Urinary Coll2-1 NO2 was also removed in the final model as it had the highest missing data (n = 261).

Logistic and linear regression models were used to describe the associations of predictors with the outcomes. The exposures (predictors of interest) are the biomarkers. Outcomes of interest are radiographic and clinical OA variables. The outcome radiographic and clinical variables were defined as K&L grade ≥ 2, JSN medial and lateral side OARSI grade of ≥ 1, osteophytes OARSI of ≥ 1, and WOMAC pain and stiffness score ≥ 3, JSW, KOOS pain and symptoms score. Logistic regression models were fitted for the following outcomes: K&L grade, JSN (medial and lateral), WOMAC (pain and stiffness) and osteophytes (medial and lateral). Odd ratios were used to describe the biomarkers of having an increase of 1 unit per exposure (outcome variables). Linear regression models were fitted for the outcome variables: JSW, KOOS pain and symptoms as the data was continuous. All models were analysed using univariate model first, where it described the association of each individual biomarker with outcome without adjusting for age, body mass index (BMI), gender and race. Fractional polynomial regression modelling was used to assess the assumption of linearity of continuous biomarkers with outcome. A full multivariable model was fitted to evaluate the association of all 19 biomarkers with the outcomes and adjusting for age, BMI, gender, and race. To demonstrate the discriminatory ability of a combination of biomarkers on predicting the radiographic and clinical outcomes, area under the curve (AUC) and receiver operator characteristic curves (ROC) was performed. Forest plots were used to provide a visualise display of the association the biomarkers with the outcome variables. The unit of measurements of some of the biomarkers were adjusted to the power of 10, 100, 1,000 and logarithmically transformed to plots the graphs.

The statistical analysis described above for baseline data were repeated for K&L grades 12 months and 24 months to check for consistency of the strength of associations across time points. Analysis of the outcome variables JSN, JSW and osteophytes, WOMAC pain and KOOS pain scores were carried out on baseline biomarker data only. In all multivariable analyses between clinical symptoms and biomarkers the data was adjusted for overall radiographic severity (K&L grade). P values of less than 0.05 was considered as statistically significant.

## Results

### Logistic regression models

Spearman correlation was used to check for the collinearity of the predictors (biomarkers) (supplementary Table 2). The results showed that serum CTXI was highly correlated with serum NTXI (r = 0.6108), urinary NTXI (r = 0.7474), urinary CTXIα (r = 0.8210) and CTXIβ (r = 0.8230) while urine NTXI was positively correlated with urinary CTXIα (r = 0.8661) and CTXIβ (r = 0.8586). Therefore, in the multivariable final regression model all predictors showing collinearity (urinary C2C, CTXIα, CTXIβ, NTXI and serum NTXI) were removed from the model and data adjusted for age, gender, BMI and race.

**Table 2:** Univariate and multivariate linear regression for JSW, KOOS pain and symptoms.

Univariate						
Biomarkers (n = 300–600)	JSW		KOOS pain		KOOS symptoms	
	Coeffecient (95% CI)	p value	Coefficient (95% CI)	p value	Coefficient (95% CI)	p value
Serum C1,2C	− 0.0002 (− 0.0070, 0.0066)	0.960	0.0079 (− 0.0634, 0.0791)	0.828	0.015 (− 0.0469, 0.077)	0.634
Serum C2C	− 0.0016 (− 0.0036, 0.0004)	0.116	− 0.0101 (− 0.0314, 0.0112)	0.353	− 0.0009 (− 0.0195, 0.0176)	0.922
Serum CPII	0.00013 (− 0.0001, 0.0005)	0.393	− 0.001 (− 0.0043, 0.0022)	0.539	− 0.0004 (− 0.0033, 0.0024)	0.769
Serum PIIANP	− 0.00001 (− 0.0002, 0.00013)	0.920	− 0.001 (− 0.0024, 0.0004)	0.148	− 0.0005 (− 0.0017, 0.0007)	0.422
Serum Coll2-1 NO2	− 0.00474 (− 0.0237, 0.0143)	0.624	− 0.1811 (− 0.3796, 0.0173)	0.074	− 0·1424 (− 0.315, 0.0303)	0.106
Serum CS846	− 0.00025 (− 0.0021, 0.0016)	0.786	− 0.0,104 (− 0.0294, 0.0086)	0.283	− 0.0138 (− 0.0303, 0.0028)	0.102
Serum MMP3	− 0.00282 (− 0.0106, 0.0049)	0.473	0.0572 (− 0.0236, 0.1379)	0.165	**0.0917 (0.0217, 0.1617)**	**0.010**
Serum CTXI	0.00006 (− 0.0004, 0.00055)	0.787	0.0022 (− 0.0029, 0.0072)	0.399	0.0004 (− 0.004, 0.0048)	0.852
Serum COMP	− 0.00189 (− 0.0360, 0.0322)	0.913	0.0,013 (− 0.0023, 0.0049)	0.480	0.0001 (− 0.003, 0.0032)	0.943
Serum HA	**− 0.00543 (− 0.0082, − 0.0026)**	**0.001**	− 0.0032 (− 0.0328, 0.0263)	0.830	0.0113 (− 0.0142, 0.0369)	0.385
Serum NTXI	0.01034 (− 0.0089, 0.0296)	0.292	0.0386 (− 0.1632, 0.2405)	0.707	− 0.0292 (− 0·2047, 0·1463)	0.744
Urine CTXII	**− 0.0105 (− 0.0157, − 0.0052)**	**0.001**	**− 0.0678 (− 0.1235, − 0.0120)**	**0.017**	**− 0.0730(− 0·1213, − 0.0247)**	**0.003**
Urine C1,2C	0.08211 (− 0.0084, 0.1726)	0.075	− 0.5382 (− 1.4914, 0.4151)	0.268	− 0·1075 (− 0·9360,0·7210)	0.799
Urine C2C	**− 0.00143 (− 0.0024, − 0.0005)**	**0.004**	0.0025 (− 0.0077, 0.0128)	0.627	− 0.0006 (− 0.0095, 0.0083)	0.889
Urine NTXI	− 0.0006 (− 0.0065, 0.0053)	0.837	0.0083 (− 0.0538, 0.0705)	0.792	0.004 (− 0.0499, 0.058)	0.884
Urine CTX1 alpha	− 0.0001 (− 0.0003, 0.00023)	0.742	0.0015 (− 0.0015, 0.0045)	0.325	0.0009 (− 0.0017, 0.0035)	0.501
Urine CTX1 beta	0.00001 (− 0.00004, 0.00008)	0.579	− 0.0001 (− 0.0007, 0.0006)	0.837	− 0.0001 (− 0.0006, 0.0005)	0.761
Urine Coll2-1 NO2	0.00023 (− 0.0085, 0.00901)	0.958	− 0.0422 (− 0.1405, 0.0561)	0.399	0.0224 (− 0.063, 0·1077)	0.607
Urine creatinine	0.01372 (− 0.0055, 0.0329)	0.161	− 0.18 (− 0.382, 0.022)	0.081	− 0.1414 (− 0·3169, 0.034)	0.114

Multivariate						
Biomarkers (n = 580)	JSW		KOOS pain		KOOS symptoms	
Serum C1,2C	0.0032 (− 0.0052, 0.0116)	0.456	0.0506 (− 0.0361, 0·1372)	0.252	0.0387 (− 0.0367, 0.1141)	0.314
Serum C2C	**− 0.0029 (− 0.0054, − 0.0004)**	**0.023**	0.0054 (− 0.0205, 0.0314)	0.681	0.0146 (− 0.008, 0.0372)	0.205
Serum CPII	**0.0005 (0.000, 0.0009)**	**0.033**	0.0012 (− 0.0031, 0.0055)	0.597	0.0014 (− 0.0024, 0.0,051)	0.468
Serum PIIANP	− 0.0001 (− 0.0002, 0.0001)	0.788	− 0.0005 (− 0.002, 0.0009)	0.466	− 0.0003 (− 0.0015, 0.001)	0.658
Serum Coll2-1 NO2	− 0.0102 (− 0.0337, 0.0133)	0.394	− 0·1316 (− 0·3743, 0·1112)	0.287	− 0·1461 (− 0·3575, 0.0652)	0.175
Serum CS846	− 0.0004 (− 0.0022, 0.0015)	0.695	− 0.0072 (− 0.0264, 0.0119)	0.459	− 0.0105 (− 0.0271, 0.0062)	0.218
Serum MMP3	− 0.0027 (− 0.0116, 0.0063)	0.559	0.0184 (− 0.0739, 0·1106)	0.696	0.0594 (− 0.0209, 0.1397)	0.147
Serum CTXI	0.0003 (− 0.0005, 0.0012)	0.415	0.0034 (− 0.0052, 0.012)	0.438	0.001 (− 0.0065, 0.0085)	0.789
Serum COMP	**0.0004 (0.0000, 0.0007)**	**0.049**	0.0015 (− 0.0023, 0.0053)	0.437	− 0.0007 (− 0.004, 0.0026)	0.665
Serum HA	**− 0.0041 (− 0.0073, − 0.0009)**	**0.013**	− 0.0017 (− 0.0349, 0.0314)	0.919	0.0057 (− 0.0232, 0.0345)	0.700
Urine CTXII	**− 0.0122 (− 0.0185, − 0.0058)**	**0.001**	**− 0.0862 (− 0.1517, − 0.0206)**	**0.010**	**− 0.0908** **(− 0.1478, − 0.0337)**	**0.002**
Urine C1,2C	0.0892 (− 0.0184, 0·1968)	0.104	− 0.0164 (− 1.1282, 1.0953)	0.977	0·3362 (− 0.6317, 1.3041)	0.495
Urine NTXI	0.0011 (− 0.0098, 0.0119)	0.848	− 0.0016 (− 0.1133, 0.1101)	0.977	0.0294 (− 0.0679, 0.1267)	0.553
Urine creatinine	0.0012 (− 0.0239, 0.0263)	0.925	− 0·1347 (− 0.3937, 0.1243)	0.307	− 0·1139 (− 0.3394, 0.1115)	0.321

Biomarkers are considered as a group in the multivariate regression model and adjusted for age, BMI, gender and race. Unit of measurements of some biomarkers (serum C1,2C, COMP, CTXI and urinary CTXII, C1,2C, Coll2-1 NO2, CTXII alpha and beta) were adjusted to the power of 10, 100, 1,000 and logarithmically transformed.

Statistically significant data are shown in bold.

In the univariable model baseline K&L grade was significantly associated with serum C2C, CPII, Coll2-1 NO2, HA and urinary CTXII, while the 12 months K&L grades correlated with serum CTXI and HA only. At 24 months K&L grades was associated with serum HA, urinary C2C and urinary CTXII (supplementary Table 3). The correlation between urinary CTXII and K&L grade were essentially similar at baseline and 24 months although the actual observed odd ratio was slightly stronger at 24 months (supplementary Table 3). In the adjusted multivariate model where, age, race, BMI and gender were controlled for, serum Coll2-1 NO2, COMP and urinary CTXII were associated with baseline K&L grade, whereas the 12 months K&L grade showed that only serum CTXI and NTXI were significant (supplementary Table 4). In the final multivariable model, only urinary CTXII had strong association with baseline and 24 months K&L grade (supplementary Table 5). As the pattern of associations of the this biomarker with K&L grades were broadly similar across all three models at baseline and 24 months, the rest of the analysis for WOMAC (pain and stiffness) JSN (medial and lateral), JSW, osteophytes (medial and lateral) modelling were carried out using baseline data only.

**Figure 2:**
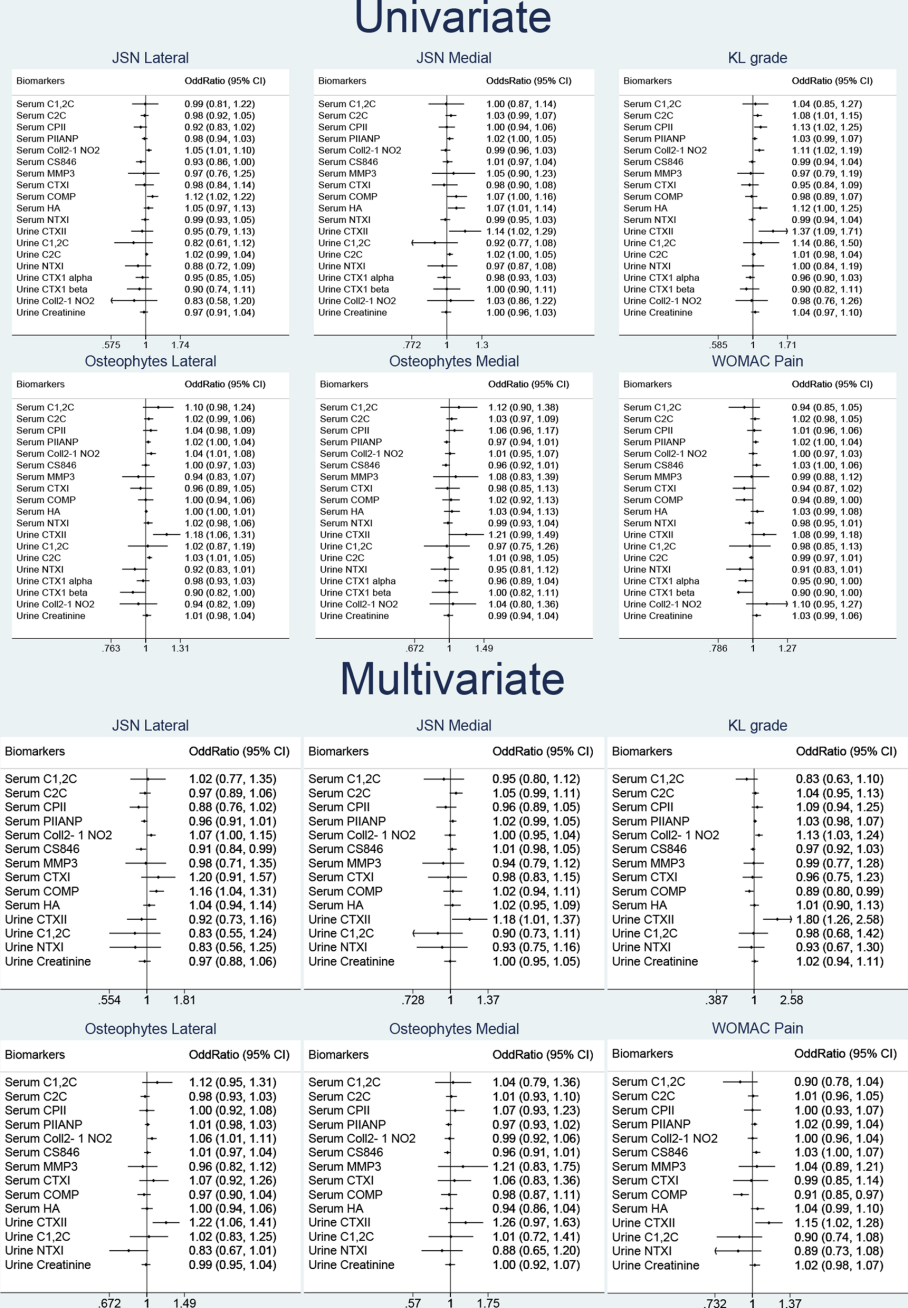
Logistic regression for baseline data. Values are odd ratios 95% confidence intervals. Unit of measurements of some biomarkers (serum C1,2C, CTXI and urinary CTXII, C1,2C, Coll2-1 NO2, CTXII alpha and beta) were adjusted to the power of 10, 100, 1,000 and logarithmically transformed. In the multivariate model biomarkers were considered as a group allowing for age, gender, BMI and race.

The results of univariable and multivariable logistic regression models are summarised in a Forest plot (Fig. 2). Of the 19 biomarkers only a handful of the markers were significant and that urinary CTXII had the strongest association compared to all other biomarkers for both radiographic and clinical outcome variables (Fig. 2). In multivariate analysis, urinary CTXII repeatedly showed positive associations with all four outcome measures used in this study; K&L grade 1.06 (95% CI 1.023–1.099), WOMAC pain 1.01 (95% CI 1.002–1.025), JSN medial 1.01 (95% CI 1.001–1.031) and osteophytes lateral 1.02 (95% CI 1.006–1.035). Serum COMP is negatively associated with KL grade and WOMAC pain. Serum COMP is negatively associated with KL grade and WOMAC pain but not JSN lateral. The results of all the biomarkers that were found to have a significant association with either radiographic and/or clinical outcomes of OA are summarised in Table 1.

**Figure 3:**
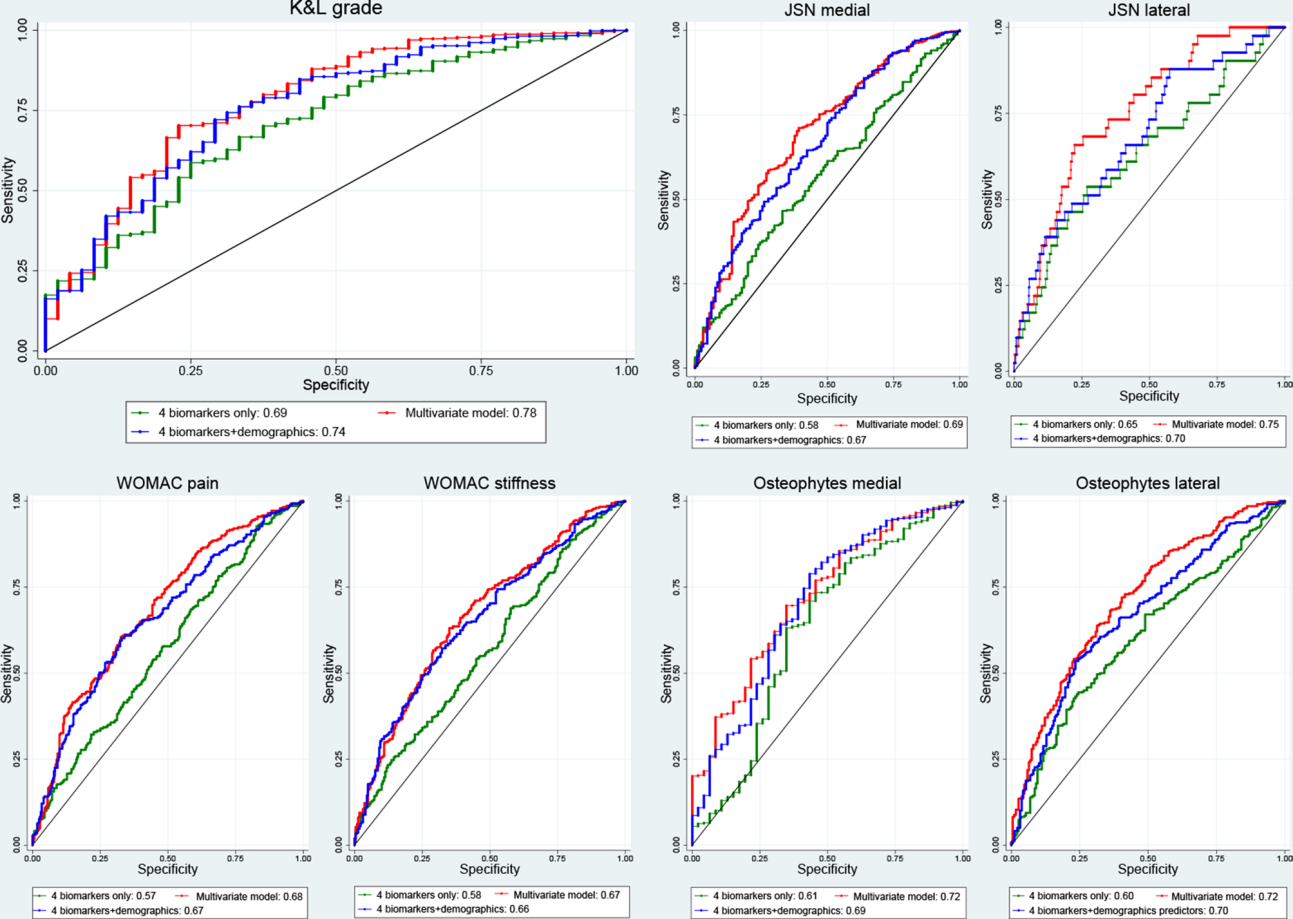
Receiver operating characteristic curves for combined biomarkers: CS846, COMP, Coll2-1 NO2 and urinary CTXII. The data was adjusted to age, BMI, gender and race. The diagonal segment is the reference line.

Urinary CTXII consistently showed strong significant associations with radiographic and clinical outcomes. The observed associations of urinary CTXII and serum Coll2-1 NO2, CS846, COMP with radiographic and clinical outcomes variables significantly improved when these 4 biomarkers were considered as a group (Fig. 3, ROC curves). For evaluation of the discriminatory diagnostic ability of the biomarkers AUC values were calculated. The combined AUC for the 4 biomarkers for K&L grade was 0.69 but when age, gender, BMI and race were included as well, it improved to 0.74 which suggest that the model was a good discriminator in predicting biomarkers with the outcome. Similarly, other radiographic and clinical variable showed improved AUC results between the 4 biomarkers combined and the multivariate model. For the 4 combined biomarkers, the AUC values for WOMAC pain, WOMAC stiffness, JSN medial, JSN lateral, osteophytes medial and osteophytes lateral were 0.57, 0.58, 0.58, 0.65, 0.61, 0.60 respectively. Whereas, the multivariate model AUC for WOMAC pain, WOMAC stiffness, JSN medial, JSN lateral, osteophytes medial and osteophytes lateral were 0.68, 0.67, 0.69, 0.75, 0.72 and 0.72 respectively (Fig. 3).

### Linear regression models

**Figure 4:**
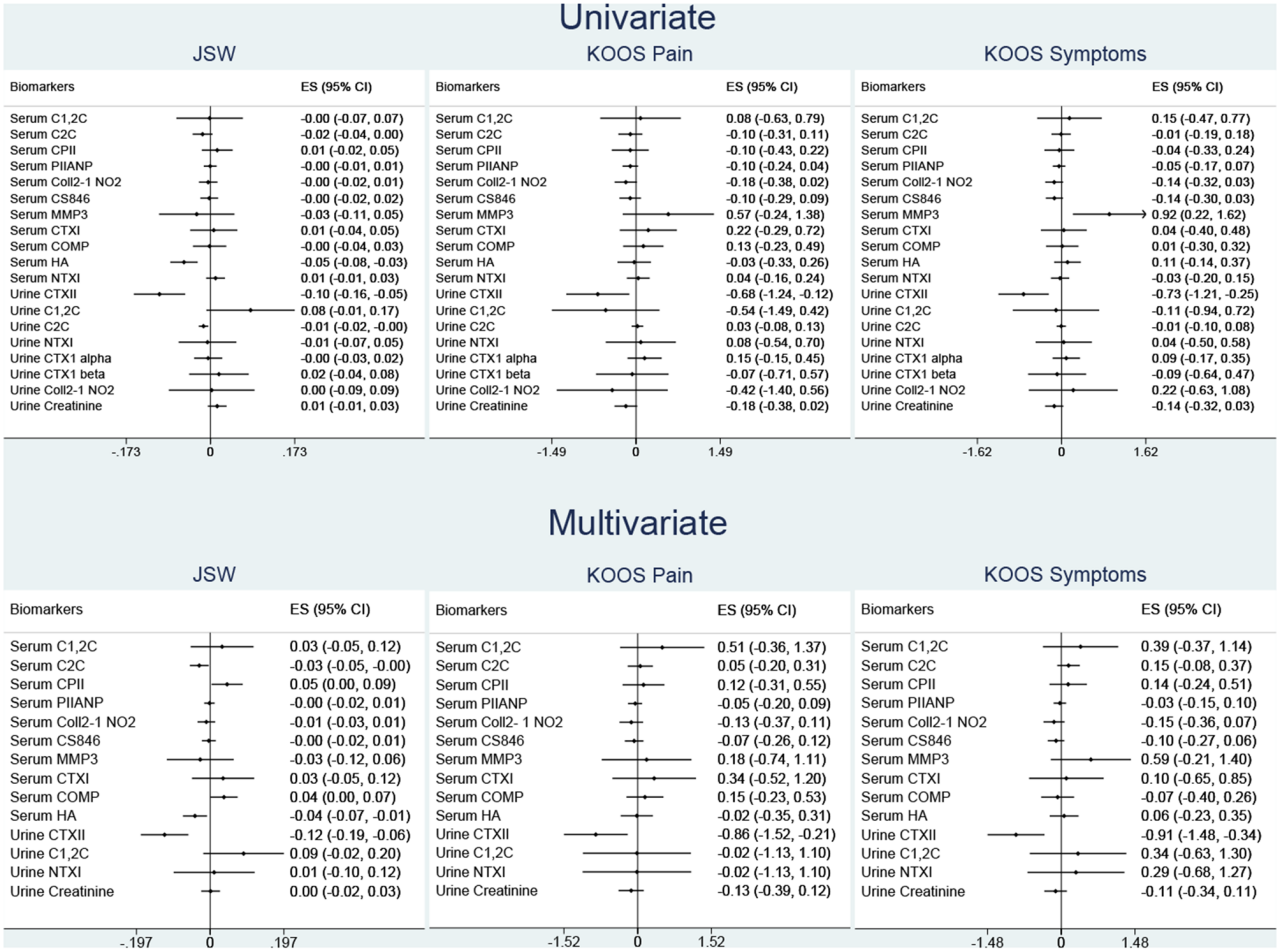
Univariate and multivariate linear regression model. Values are coefficient 95% confidence intervals. Unit of measurements of some biomarkers (serum C1,2C, COMP, CTXI and urinary CTXII, C1,2C, Coll2-1 NO2, CTXII alpha and beta) were adjusted to the power of 10, 100, 1,000 and logarithmically transformed. Biomarkers are considered as a group in the multivariate regression model and adjusted for age, BMI, gender and race.

Linear regression was used to determine the association of the biomarkers with JSW, KOOS pain and symptoms. The repeated measures showed that serum HA (p: < 0.01), urinary C2C (p: 0.04) and CTXII (p: < 0.01) are associated with JSW in the univariate model. However, in the multivariate model, serum C2C, CPII, COMP, HA and urinary CTXII showed significant association (p: < 0.01 for all 5 biomarkers) with JSW (Table 2). Urinary CTXII was associated with both KOOS pain and symptoms across the different models; univariate and multivariate (Fig. 4).

In both multivariate logistic and linear regression of clinical symptoms (KOOS pain and symptoms, WOMAC pain and stiffness) and biomarkers data was adjusted for the radiographic severity (K&L grades). The results showed no differences in the AUC values for WOMAC pain and WOMAC stiffness. For WOMAC pain AUC unadjusted and adjusted were 0.67 and 0.68 respectively. For WOMAC stiffness unadjusted and adjusted AUC were 0.66 and 0.67 respectively. Similarly, when adjusting for the radiographic severity in the multivariate linear regression (KOOS pain and KOOS symptoms), the results showed that only urinary CTXII was significant. the values allowing for radiographic severity were − 0.0775037 (− 0.1439451, − 0.0110622), p: 0.022 [coefficient, 95% CI, and p values]. The results were very similar to the previous multivariate linear regression model without adjusting for radiographic severity (Table 2).

## Discussion

OA is the most common joint disease and early diagnosis and monitoring of progression of the disease using biomarkers have gained considerable attention in recent years. However, despite extensive investigation, there are currently no biomarkers approved by the FDA for diagnosis and monitoring of OA due to limited specificity and sensitivity. In this study, we have used one of the largest publicly available datasets to identify potentially clinically useful biomarkers for OA. Our data show that of the 19 biomarkers only 4 (serum Coll2-1 NO2, CS846, COMP and urinary CTXII) were consistently associated with established radiographic and/or clinical features of OA (Table 1). Our results also show that urinary CTXII had the strongest and most consistent associations with radiographic and clinical features of OA. These biomarkers are independent of one another and provide additional predictive power over and above established demographic predictors of OA such as age, gender, BMI and race. Therefore, we suggest that these 4 biomarkers are the most promising biomarkers for investigating of OA and may be considered clinically useful particularly urinary CTXII. Identification of these 4 biomarkers as clinically useful markers will help to focus future OA studies and is a crucial step in the realization of individualized care for patients with OA.

The results show that urinary CTXII is significantly associated with K&L grades, osteophytes scores on the medial site, WOMAC (stiffness), JSW, and KOOS (pain). Urinary CTXII has been investigated extensively and shown to be elevated in OA patients with radiographic OA^19,20^, associated with pain, K&L grades and other radiographic features of OA, and predicts radiographic pain progression^21–23^. The Joseph et al.^24^ and Kraus et al.^25^ studies used data from the OAI cohort but in both studies the presence of bilateral OA could have influenced the results since patient reported pain on physical function and pain score were used^24,25^. In this study, we have chosen the worst affected knee and patient-based measurements of urinary CTXII and other biomarkers are therefore more likely to be a better reflection of the disease process in individual patients. Moreover, the Joseph et al. study was focused on correlation of the biomarkers with MRI T2 measures of cartilage matrix degeneration suggesting that biomarkers (serum MMP3, serum HA, serum COMP) are sensitive to cartilage changes, while the Kraus et al. study investigated short-term (2 years) prognostic value of 18 of the 19 biomarkers^24,25^. The latter study demonstrated that several of these biomarkers (serum HA, NTXI, CTXI and urinary CTXII, C2C, NTXI, CTXI alpha CTXI beta) could predict worsening of pain and radiographic OA over 2 years^24^. Our study is uniquely designed to identify biomarkers that would be of clinical value for early decision making about diagnosis have led to identification of different biomarker combination (serum Coll2-1 NO2, CS846, COMP and urinary CTXII) than those identified by the latter studies using the same cohort.

In our earlier studies of urinary CTXII we found that CTXII was significantly higher in the progressors (defined as either a reduction of the tibiofemoral joint space width by ≥ 2 mm or total knee replacement surgery for either knee occurring during the 5-year follow-up) and remained high throughout the study period^12^. Moreover, there was a rapid rise in CTXII during the first 2 years of follow-up in the progressors indicating a more aggressive course of the disease in these patients at an early stage^12^. The results of the current study are broadly in agreement with previous studies, and additionally suggest that unlike any of the other current biomarkers, urinary CTXII is associated with the main radiographic features of OA (JSN, osteophytes and K&L grade) as well as clinical outcome variables WOMAC stiffness and a knee specific measure of OA pain (KOOS score). In the multivariable analyses between these clinical symptoms and the biomarker adjusting for radiographic severity gave almost identical AUC values suggesting that the association observed are independent of radiographic severity as measured by K&L grade. Another new observation reported in this study is the association of urinary CTXII with JSN on the medial compartment of the knee joint. This is important since both cartilage degradation and bone changes usually occur on the medial side first^26,27^ and therefore CTXII may be a marker of early cartilage loss as well as bone remodelling.

It is generally accepted that there is a high discordance between clinical and radiographic knee OA^28–30^. Thus, only about 50% of the patients with radiographic knee OA have knee pain^29^, and even levels of disability experienced by patients appear to correlate better with their age and psychological involvement than with their radiographic scores^28,30^. Despite the widely reported discordance between radiographic damage and pain, we show here that urinary CTXII reflects knee specific pain in OA as we well as radiographic evidence of joint damage. Therefore, we suggest that urinary CTXII may aid in early diagnosis of OA in symptomatic patients without radiographic evidence of OA. Of-course, further studies in patients with symptomatic OA is needed to confirm the diagnostic and clinical usefulness of urinary CTXII. In addition, the data reported here are from an aging OA cohort, therefore further studies in independent younger cohort, post-traumatic OA and metabolic OA are needed to confirm the clinical usefulness of CTXII.

Serum COMP is a pentameric non-collagenous glycoprotein belonging to the heterogeneous family of thrombospondin which can bind to collagen type I, II, and IX. It functions as a catalyst in collagen fibril formation and many studies have shown that serum COMP is elevated in OA and after knee injury^31,32^. Serum COMP has been extensively studied by our group and others and shown to have some value as a diagnostic and/or prognostic maker of knee OA^32,33^. Most of the previous studies investigating serum COMP have shown that serum COMP levels reflect structural and metabolic changes in OA^34,35^. A few studies that have looked for associations with clinical symptoms have shown inconsistent results^36^. The data presented here is from one of the largest and best characterised cohort of OA patients and shows that serum COMP is associated with two of the main features of structural change in OA (JSN and K&L grade) as well as joint pain. The association of serum COMP with structural damage in OA is easy to explain as it is a marker of cartilage damage, but its association with joint pain is not straightforward. Synovium is a likely source of joint pain in OA and since high COMP levels are also found in synovium, COMP levels may reflect low-grade synovial inflammation in knee OA. This is consistent with data showing elevated serum COMP in patients with RA, a disease characterised by synovial inflammation^37^. Other investigators have suggested that urinary CTXII and serum COMP reflect the metabolism of type II collagen fibres^38^ and are both associated with cartilage degradation. Therefore, serum COMP is one of the better biomarkers of OA which reflect structural damage as well as main joint symptom, pain. However, it is worth noting that serum COMP is not OA-specific and elevated levels have been reported in other chronic diseases^37,39^.

CS846 is a chondroitin sulphate epitope considered to be a marker of aggrecan degradation and elevated levels have been reported by many studies following joint injury^40,41^. Earlier studies have shown that CS846 was almost absent from normal adult cartilage, but increased levels have been reported in OA^42,43^ and therefore this marker may be considered to have some degree of disease specificity. Serum COMP and CS846 are thought to be released following initial damage and activation of repair mechanism and as such may reflect pathological increase in turnover of newly formed matrix^44,45^. A number of previous studies analysed serum COMP and CS846 in OA and found the two biomarkers are positively correlated, and when combined the biomarkers gave significantly improved AUC values compared to that of the single biomarker for diagnosis of OA^46^. Our data show that serum CS846 was associated with WOMAC stiffness and JSN and but it was not correlated with serum COMP. We suggest that the damage to type II collagen molecule is the key trigger for release and elevated levels of both serum COMP and CS846 in OA. This hypothesis would be consistent with our observation that urinary CTXII (a specific marker of type II collagen degradation), which is known to be elevated in OA, appear to be a marker of both structural damage and symptoms but did not correlate directly with serum COMP or CS846 in this study.

The association of serum COMP and serum CS846 with joint space narrowing on the lateral side was an unexpected finding because as markers of cartilage degradation serum levels of both markers were expected to correlate with loss of cartilage irrespective of joint sides. One possible explanation is that serum COMP and CS846 are derived from different joint compartments including synovium and meniscus and the association seen may represent type 1 error.

Coll2-1NO2 is the nitrated form of Coll2-1 peptide (108HRGYPGLDG116) found in the triple helix of the type II collagen molecule^47^. Unlike the other biomarkers of OA, Coll2-1NO2 is a relative new marker of OA and there is only a limited number of studies of this marker compared to CTXII, COMP and CS846. Coll2-1 NO2 is thought to be produced by stressed chondrocytes following cartilage damage and activation of inflammatory cytokines^48^. Serum Coll2-1 NO2 is elevated in OA subjects and urinary Coll 2-1 NO2 levels have been reported to be associated with global WOMAC score and WOMAC pain and predictive of radiographic knee OA^49^. Our data showing association of serum Coll2-1 NO2 with osteophytes and K&L grades is therefore broadly in agreement with published data and additionally suggest Coll2-1 NO2 is a marker of overall radiographic severity of knee OA.

Our study has identified four clinically useful biomarkers for OA of which only urinary CTXII was consistently correlated with the main radiographic features of OA and symptoms. Identification of these biomarkers will help future studies to focus on these markers instead of a long list of markers many which are not associated with clinical and/or radiographic features of OA. The AUC for the prediction of individual markers with the association of K&L grade ≥ 2 are ranging from 0.4 to 0.6 which means that each individual markers are a poor diagnostic discriminatory ability (Supplementary Table 3). However, when the 4 biomarkers are combined together adjusted for age, BMI, gender and race, the predictive ability increases to 0.74 (Fig. 3), suggesting that a clinically useful biomarker may be created for better specificity and sensitivity for diagnosing and monitoring OA. Similar results were obtained when the repeated measures were analysed with worse knee side measurements of JSN medial and lateral side (OARSI score: ≥ 1), WOMAC pain and stiffness (score: ≥ 3) and osteophytes in medial and lateral sides (OARSI score: ≥ 1) providing further support to the contention that a combination of biomarkers can improve prediction of clinical and radiographic severity of OA. The severity of radiographic, clinical features and the demographic data provide a clear evidence that development of OA does not depend on a single factor and that a combination of biomarkers may be created to investigate OA pathology^46^. Our data provided clear evidence for this and is consistent with results of a recent study where the authors showed that combining demographic factors, and K&L grade with urinary CTXII significantly improved the AUC for prediction of total joint replacement over a two year period^50^.

The strengths of the study included comprehensive in-depth analysis of the largest cohort of patients with a wide range of urine and serum biomarkers, and well-defined clinical symptoms and radiographic OA outcomes. In this study the biomarkers were measured in serum and urine, presumably reflecting release of the biomarkers from all joints (whether symptomatic or asymptomatic) therefore the associations between markers and radiographic and/or clinical features of OA in the index joint may be even stronger than that is reported here.

There are several limitations of this study. We had to exclude the urinary Coll2-1 N02 due to missing data, and there was collinearity between some of the biomarkers (supplementary Table 2) which meant we had to exclude biomarkers from the final adjusted model. Biomarkers are patient level variable, and do not distinguish between right and left knees, so we made the decision to choose the worst affect knee for OA outcomes which were collected for both sides. The associations observed are cross sectional in nature (data on biomarkers and outcomes collected all at baseline), and these findings do not inform as to the predictive validity of biomarkers on disease progression. Data are observational and represent associations and causality cannot be inferred. Despite these limitations, our study has identified 4 high value biomarkers of which urinary CTXII may be considered as clinically useful for investigation of OA.

In conclusion, of the 19 biomarkers analysed urinary CTXII, serum COMP, CS846 and Coll2-1 NO2 appear to be the most promising biomarkers for OA as they relate to both structural damage as well as symptoms in knee OA. These markers do not correlate with each other but when combined the AUC values improved compared to that of the single biomarker. Our data show that Urinary CTXII had the strongest and consistent associations with clinical symptoms of OA as well as radiographic evidence of joint damage suggesting that urinary CTXII is the best-qualified biomarker for evaluation of OA. We suggest that urinary CTXII may be a clinically useful biomarker and it would be highly relevant for care planning and for development of disease modifying drugs for this very common condition.

## Supplementary information

Supplementary file1 (PDF 308 kb)
