# Supplementary material for: Multivariable logistic and linear regression models for identification of clinically useful biomarkers for osteoarthritis
Source: Sci Rep. 2020 Jul 9;10:11328. doi: 10.1038/s41598-020-68077-0 (PMC7347626; doi:10.1038/s41598-020-68077-0)
Supplement: Supplementary file 1 — Supplementary file1 (PDF 308 kb) [file 41598_2020_68077_MOESM1_ESM.pdf]

Supplementary Dataset

Clinically useful biomarker in osteoarthritis (OA): urinary CTXII is the best-qualified marker for evaluation of OA?

Yulia Liem[1], Andy Judge[1], John Kirwan[2], Khadija Ourradi[1], Yunfei Li[1] and Mohammed Sharif[1*]

Corresponding Author:
*Dr Mohammed Sharif
Translational Health Sciences
Bristol Medical School
Musculoskeletal Research Unit
University of Bristol
Learning and Research Building (Level 2)
Southmead Hospital
Bristol
BS10 5NB
United Kingdom
Contact: +44 (0) 117 41 47926, Email: mo.sharif@bristol.ac.uk

Affiliations:
[1]Translational Health Science, Bristol Medical School, Musculoskeletal Research Unit, Learning & Research, Southmead Hospital, University of Bristol, Bristol, United Kingdom
[2]Emeritus Professor at University of Bristol

Age	Frequencies	Percentage (%)
40 - 50	510	11·36
50 - 60	1566	34·87
60 - 70	1369	30·48
70 - 80	1046	23·29
Total	*4491*	*100*
**Gender**	**Frequencies**	**Percentage (%)**
Male	1881	41·88
Female	2610	58·12
Total	*4491*	*100*
**Race**	**Frequencies**	**Percentage (%)**
White / Caucasian	3600	80·16
Black / African American	780	17·37
Asian	38	0·85
Others	69	1·54
Missing	4	0·09
Total	*4491*	*100*
**Body Mass Index**	**Frequencies**	**Percentage (%)**
Underweight	11	0·24
Normal	1069	23·8
Overweight	1764	39·28
Class I Obese	1178	26·23
Class II Obese	392	8·73
Class II Obese	77	1·71
Total	*4491*	*100*

Supplementary table 1. Demographics data of the entire OAI cohort

**Biomarkers**		**Serum**											**Urine**							
		**C12C**	**C2C**	**CPII**	**PIIANP**	**Coll2-1 NO2**	**CS846**	**MMP3**	**CTXI**	**COMP**	**HA**	**NTXI**	**CTXII**	**C12C**	**C2C**	**NTXI**	**CTX1 alpha**	**CTX1 beta**	**Coll2-1 NO2**	**Creatinine**
**Serum**	**C12C**	1·0000																		
	**C2C**	0·4028	1·0000																	
	**CPII**	0·3880	0·3772	1·0000																
	**PIIANP**	0·0849	0·1239	0·0715	1·0000															
	**Coll2-1 NO2**	0·2812	0·3547	0·3783	-0·0249	1·0000														
	**CS846**	0·0052	-0·0029	-0·0477	-0·0644	0·0047	1·0000													
	**MMP3**	-0·0280	-0·2263	-0·2100	-0·1038	-0·1641	-0·0250	1·0000												
	**CTXI**	0·0599	0·0219	-0·0232	0·0099	-0·0005	0·1719	-0·0517	1·0000											
	**COMP**	0·0913	0·1218	0·1248	0·0720	0·0848	-0·0170	0·1963	0·0475	1·0000										
	**HA**	0·0653	0·0570	0·0099	-0·0598	0·0391	-0·0618	0·1316	0·0696	0·2707	1·0000									
	**NTXI**	-0·1154	-0·0869	-0·0508	0·0170	0·0402	0·1082	-0·0131	0·6108	0·0938	0·1200	1·0000								
**Urine**	**CTXII**	0·1244	0·1637	0·0756	0·0773	0·1690	-0·0728	-0·1627	0·3026	0·1043	0·3274	0·2254	1·0000							
	**C12C**	-0·0378	0·0577	-0·0562	-0·1576	0·0072	0·0432	-0·0189	-0·0751	0·0053	-0·0284	-0·1021	-0·0272	1·0000						
	**C2C**	0·1332	0·1241	0·0559	0·0671	0·1220	-0·0454	-0·0323	0·1173	0·1389	0·4113	0·1072	**0·6991**	-0·0263	1·0000					
	**NTXI**	0·0586	0·0880	-0·0153	-0·0133	0·0719	0·1330	-0·2207	**0·7474**	-0·0187	0·1099	0·4949	0·4113	0·1359	0·2815	1·0000				
	**CTX1 alpha**	0·0783	0·1069	0·0303	0·0330	0·0605	0·1570	-0·1768	**0·8210**	-0·0270	0·1009	0·5364	0·4190	0·0089	0·2739	**0·8661**	1·0000			
	**CTX1 beta**	0·0563	0·0602	-0·0335	0·0083	0·0437	0·1891	-0·2118	**0·8230**	-0·0816	0·0255	0·5058	0·3203	0·0834	0·1452	**0·8586**	**0·8991**	1·0000		
	**Coll2-1 NO2**	0·0683	0·0190	0·0871	-0·0689	-0·0318	-0·0947	-0·0573	-0·0597	0·0832	0·0790	-0·0758	0·1007	0·3069	0·1674	0·1526	-0·0004	0·0262	1·0000	
	**Creatinine**	-0·0940	-0·0139	-0·1245	-0·0467	-0·0955	0·0653	0·0985	0·0645	0·0338	-0·0359	0·0223	-0·0523	0·2843	-0·2706	-0·1185	-0·0749	0·0310	-0·0812	1·0000

Supplementary table 2· Spearman correlations between biomarkers for checking collinearity at baseline.

Univariate models (K&L grade)									
N=300 - 600	**Baseline**			**12 Months**			**24 Months**		
	Odd Ratio (95% CI)	P value	AUC	Odd Ratio (95% CI)	P value	AUC	Odd Ratio (95% CI)	P value	AUC
Serum C12C	1·0040 (0·9841, 1·0244)	0·696	0·506	0·9957 (0·9754, 1·0164)	0·679	0·536	0·9974 (0·9760, 1·0192)	0·813	0·498
Serum C2C	**1·0075 (1·0009, 1·0142)**	**0·025**	**0·597**	1·0019 (0·9949, 1·0089)	0·601	0·511	0·9999 (0·9934, 1·0064)	0·972	0·522
Serum CPII	**1·0012 (1·0002, 1·0022)**	**0·024**	**0·599**	1·0000 (0·9990, 1·0010)	0·949	0·486	1·0003 (0·9993, 1·0013)	0·569	0·528
Serum PIIANP	1·0003 (0·9999, 1·0007)	0·150	0·569	1·0004 (0·9999, 1·0009)	0·094	0·578	1·0115 (0·9508, 1·0761)	0·718	0·505
Serum Coll21NO2	**1·1054 (1·0246, 1·1926)**	**0·010**	**0·607**	1·0563 (0·9778, 1·1412)	0·165	0·558	1·0115 (0·9508, 1·0761)	0·718	0·487
Serum CS846	0·9989 (0·9939, 1·0039)	0·660	0·507	0·9978 (0·9926, 1·0029)	0·392	0·587	0·9997 (0·9938, 1·0056)	0·920	0·511
Serum MMP3	0·9968 (0·9762, 1·0177)	0·759	0·521	1·0111 (0·9776, 1·0458)	0·520	0·520	1·0019 (0·9733, 1·0313)	0·899	0·486
Serum CTXI	0·9995 (0·9982, 1·0009)	0·506	0·492	**0·9984 (0·997, 0·9998)**	**0·029**	**0·550**	0·9989 (0·9975, 1·0003)	0·133	0·546
Serum COMP	0·9998 (0·9988, 1·0007)	0·651	0·513	1·0002 (0·9991, 1·0012)	0·770	0·518	0·9999 (0·9988, 1·0009)	0·808	0·499
Serum HA	**1·0114 (1·0002, 1·0228)**	**0·046**	**0·623**	**1·0155 (1·0012, 1·0299)**	**0·034**	**0·630**	**1·0184 (1·0035, 1·0335)**	**0·015**	**0·656**
Serum NTXI	0·9872 (0·9369, 1·0401)	0·628	0·502	1·0301 (0·9586, 1·107)	0·419	0·534	1·0284 (0·9580, 1·1039)	0·440	0·545
Urine CTXII	**1·0317 (1·0090, 1·0550)**	**0·006**	**0·671**	1·0225 (0·9996, 1·0458)	0·054	0·635	**1·0335 (1·0070, 1·0607)**	**0·013**	**0·655**
Urine C12C	1·1367 (0·8600, 1·5024)	0·368	0·546	1·3294 (0·9633, 1·8347)	0·083	0·574	1·1437 (0·8240, 1·5875)	0·422	0·536
Urine C2C	1·0012 (0·9980, 1·0043)	0·475	0·591	1·0030 (0·9987, 1·0073)	0·173	0·614	**1·0074 (1·0022, 1·0126)**	**0·005**	**0·649**
Urine NTXI	1·0003 (0·9832, 1·0177)	0·970	0·519	0·9895 (0·9739, 1·0054)	0·194	0·494	0·9938 (0·9727, 1·0154)	0·573	0·493
Urine CTX1alpha	0·9996 (0·9989, 1·0003)	0·240	0·479	0·9996 (0·9987, 1·0005)	0·365	0·475	0·9996 (0·9985, 1·0006)	0·444	0·481
Urine CTX1beta	0·9999 (0·9998, 1·0001)	0·379	0·532	0·9999 (0·9997, 1·0000)	0·138	0·524	0·9999 (0·9997, 1·0001)	0·456	0·510
Urine Coll21NO2	0·9981 (0·9735, 1·0234)	0·885	0·456	0·9846 (0·9693, 1·0002)	0·053	0·607	0·9980 (0·9696, 1·0272)	0·891	0·442
Urine Creatinine	1·0369 (0·9749, 1·1029)	0·250	0·554	1·0374 (0·9703, 1·1091)	0·282	0·538	1·0439 (0·9687, 1·1250)	0·260	0·561

Supplementary table 3 Univariate logistic regression model for biomarker associations with KL grade at Baseline, 12 months and 24 months. Unit of measurements of some biomarkers (serum C12C, COMP, CTXI and urinary CTXII, C12C, Coll2-1 NO2, CTXII alpha and beta) were adjusted to the power of 10, 100, 1000 and logarithmicall

Adjusted Multivariate (K&L grade)									
n=546	Baseline			12 Months			24 Months		
	Odd Ratio (95% CI)	P value	AUC	Odd Ratio (95% CI)	P value	AUC	Odd Ratio (95% CI)	P value	AUC
Serum C12C	0·9812 (0·954, 1·0093)	0·187	0·7980	0·97976 (0·95078, 1·00963)	0·182	0·7800	0·97103 (0·93788, 1·00536)	0·097	0·8234
Serum C2C	1·0048 (0·996, 1·0137)	0·282		1·00126 (0·99153, 1·01109)	0·801		1·00486 (0·99401, 1·01581)	0·382	
Serum CPII	1·0008 (0·9994, 1·0022)	0·275		1·00005 (0·99874, 1·00136)	0·943		1·00104 (0·99925, 1·00282)	0·255	
Serum PIIANP	1·0003 (0·9998, 1·0007)	0·257		1·00038 (0·99987, 1·00090)	0·145		1·00016 (0·99961, 1·00071)	0·563	
Serum Coll21NO2	**1·1340 (1·0298, 1·2488)**	**0·011**		1·09907 (0·98637, 1·22464)	0·087		**1·12359 (1·00045, 1·26188)**	**0·049**	
Serum CS846	0·9977 (0·9917, 1·0038)	0·462		0·99723 (0·9909, 1·00361)	0·394		0·99568 (0·98885, 1·00255)	0·217	
Serum MMP3	0·9982 (0·9728, 1·0243)	0·894		0·99743 (0·95914, 1·03725)	0·898		0·99642 (0·96548, 1·02834)	0·823	
Serum CTXI	1·0009 (0·9979, 1·0038)	0·566		**0·99600 (0·99262, 0·99938)**	**0·020**		1·00081 (0·99712, 1·00451)	0·668	
Serum COMP	**0·9987 (0·9976, 0·9999)**	**0·029**		0·99935 (0·99811, 1·00060)	0·311		**0·99829 (0·99695, 0·99963)**	**0·013**	
Serum HA	1·0012 (0·9898, 1·0127)	0·84		1·01066 (0·99274, 1·02890)	0·246		1·01503 (0·99574, 1·03471)	0·128	
Serum NTXI	0·9948 (0·9285, 1·0658)	0·882		**1·13945 (1·00703, 1·28929)**	**0·038**		1·01946 (0·92198, 1·12724)	0·707	
Urine CTXII	**1·0700 (1·0270, 1·1148)**	**0·001**		1·01889 (0·98335, 1·05571)	0·302		**1·06842 (1·01538, 1·12424)**	**0·011**	
Urine C12C	0·9731 (0·6612, 1·4322)	0·89		1·45672 (0·95100, 2·23136)	0·084		0·94022 (0·58042, 1·52308)	0·802	
Urine C2C	0·9985 (0·9938, 1·0032)	0·528		1·00033 (0·99467, 1·00603)	0·909		0·99837 (0·99228, 1·00451)	0·602	
Urine NTXI	1·0219 (0·9674, 1·0795)	0·439		0·97537 (0·93669, 1·01565)	0·227		1·03897 (0·96960, 1·11330)	0·278	
Urine CTX1alpha	0·9985 (0·9960, 1·0010)	0·238		1·00192 (0·99924, 1·00460)	0·161		0·99799 (0·99477, 1·00123)	0·224	
Urine CTX1beta	0·9998 (0·9995, 1·0002)	0·314		0·99989 (0·99948, 1·00031)	0·610		0·99966 (0·99921, 1·00011)	0·136	
Urine Creatinine	1·0155 (0·9318, 1·1067)	0·726		0·98874 (0·90873, 1·07578)	0·792		0·98688 (0·89406, 1·08933)	0·793	

Supplementary table 4 Multivariate model for biomarker associations with KL grade at baseline, 12 months and 24 months· Unit of measurements of some biomarkers (serum C12C, COMP, CTXI and urinary CTXII, C12C, Coll21 NO2, CTXII alpha and beta) were adjusted to the power of 10, 100, 1000 and logarithmically transformed· The data was adjusted for age, BMI, gender and race. Urinary Coll2-1 NO2 was removed due to missing data.

Final multivariate model (K&L grade)									
n=547	Baseline			12 Months			24 Months		
	Odd Ratio (95% CI)	P value	AUC	Odd Ratio (95% CI)	P value	AUC	Odd Ratio (95% CI)	P value	AUC
Serum C12C	0·9816 (0·9546, 1·0093)	0·191	0·7856	0·97930 (0·95108, 1·00835)	0·161	0·775	0·97174 (0·93924, 1·00535)	0·098	0·8052
Serum C2C	1·0036 (0·9951, 1·0121)	0·407		0·99983 (0·99074, 1·00899)	0·970		1·00285 (0·9925, 1·0133)	0·591	
Serum CPII	1·0008 (0·9994, 1·0023)	0·254		0·99987 (0·9986, 1·00114)	0·837		1·00108 (0·99933, 1·00284)	0·227	
Serum PIIANP	1·0003 (0·9998, 1·0007)	0·252		1·00041 (0·9999, 1·00092)	0·118		1·00019 (0·99965, 1·00074)	0·488	
Serum Coll21NO2	**1·1316 (1·0289, 1·2445)**	**0·011**		**1·11319 (1·00225, 1·23641)**	**0·045**		1·11904 (0·99887, 1·25367)	0·052	
Serum CS846	0·9972 (0·9913, 1·0032)	0·364		0·99818 (0·99203, 1·00437)	0·564		0·99531 (0·98865, 1·00201)	0·17	
Serum MMP3	0·9992 (0·9737, 1·0253)	0·950		1·00051 (0·96272, 1·03978)	0·979		0·99938 (0·96843, 1·03133)	0·969	
Serum CTXI	0·9996 (0·9971, 1·0021)	0·761		0·99813 (0·99569, 1·00057)	0·133		0·99929 (0·99627, 1·00232)	0·645	
Serum COMP	**0·9988 (0·9977, 0·9999)**	**0·036**		0·99940 (0·99818, 1·00062)	0·335		**0·99837 (0·99706, 0·99969)**	**0·015**	
Serum HA	1·0011 (0·9898, 1·0125)	0·851		1·00953 (0·99236, 1·02700)	0·279		1·01517 (0·99577, 1·03494)	0·126	
Urine CTXII	**1·0607 (1·0231, 1·0996)**	**0·001**		1·02424 (0·99375, 1·05566)	0·120		**1·05914 (1·01327, 1·1071)**	**0·011**	
Urine C12C	0·9837 (0·6802, 1·4226)	0·930		1·34530 (0·8794, 2·05804)	0·171		0·94176 (0·59899, 1·48067)	0·795	
Urine NTXI	0·9933 (0·9609, 1·0268)	0·691		0·99595 (0·96468, 1·02823)	0·803		0·9939 (0·9544, 1·03503)	0·767	
Urine Creatinine	1·0208 (0·9381, 1·1107)	0·633		0·98596 (0·90896, 1·06949)	0·733		0·99452 (0·9024, 1·09604)	0·912	

Supplementary table 5. Multivariate logistic regression model for biomarker associations with KL grade at baseline, 12 months and 24 months. Unit of measurements of some biomarkers (serum C12C, COMP, CTXI and urinary CTXII, C12C, Coll21 NO2, CTXII alpha and beta) were adjusted to the power of 10, 100, 1000 and logarithmically transformed· The data was adjusted for age, BMI, gender and race.
